# Supplementary material for: CD4 and CD8 co-receptors modulate functional avidity of CD1b-restricted T cells
Source: Nat Commun. 2022 Jan 10;13:78. doi: 10.1038/s41467-021-27764-w (PMC8748927; doi:10.1038/s41467-021-27764-w)
Supplement: Supplementary file 1 — Supplementary Information [file 41467_2021_27764_MOESM1_ESM.pdf]

SUPPLEMENTARY INFORMATION

Cohort	ID	IGRA Result	TST Result	Age	Sex
SAC	SAC1011	N/A	N/A	N/A	N/A
SAC	SAC212671	N/A	N/A	N/A	N/A
SAC	SAC218703	N/A	N/A	N/A	N/A
SAC	SAC246764	N/A	N/A	N/A	N/A
SAC	SAC283958	N/A	N/A	N/A	N/A
ACS	01-0676	Negative	0	15	M
ACS	11-0116	Negative	0	14	F
ACS	11-0117	Negative	0	14	M
ACS	11-0178	Negative	0	13	F
ACS	01-0678	Negative	0	15	M
ACS	03-0548	Positive	15.1	12	M
ACS	09-0210	Positive	17	14	M
ACS	11-0097	Positive	12	18	M
ACS	03-0707	Positive	13	15	M
ACS	01-0774	Positive	15	12	F

Supplementary Table 1. Human samples used for *ex vivo* analysis of SGL-specific T cells. Human samples used in this study are listed by cohort of origin and participant identifier (ID). Seattle Assay Controls (SAC) are healthy donors from Seattle, WA, that were enrolled through the HIV Vaccine Trials Network (HVTN). The Adolescent Cohort Study (ACS) is composed of M.tb-infected or M.tb-uninfected adolescents from South Africa. Interferon-γ release assay (IGRA) and tuberculin skin test (TST) results are listed and concordantly positive or negative[1]. TST induration is reported here in mm. Age reflects the age at last birthday at the time the sample was collected. Sex is self-reported.

Specificity	Purpose	Fluorochrome	Clone	Supplier	Dilution
CD3	Lineage	BUV395	UCHT1	BD Biosciences	1:50
CD4	Lineage	APC H7	13B8.2	BD Biosciences	1:50
CD8β	Lineage	BB700	2ST8.5H7	BD Biosciences	1:10
CD45RA	Memory	BUV737	HI100	BD Biosciences	1:500
CCR7	Memory	BV711	150503	BD Biosciences	1:100
Pan-γδ	γδ T cells	PE-Vio770	11F2	Miltenyi Biotec	1:50
Vδ2	Vγ9δ2 T cells	AF700	B6	BioLegend	1:100
TRAV1-2	TCR Identification	BV605	3C10	BioLegend	1:25
CD14	Exclusion	V785	M5E2	BioLegend	1:50
CD19	Exclusion	V785	SJ25C1	BioLegend	1:50
Fixable Green Dead Cell Stain	Viability	FITC	N/A	Life Technologies	1:1000
CD1b-SGL	M.tb lipid antigen-specific T cells	ECD	N/A	Custom	1:25
CD1b-SGL	M.tb lipid antigen-specific T cells	PE	N/A	Custom	1:25
CD1b-Mock	Exclusion	V510	N/A	Custom	1:25

Supplementary Table 2. Flow cytometry panel used for *ex vivo* identification of SGL-specific T cells.

		Tetramer-Positive		Bulk T cells		
V Gene	Antigen	Positive	Negative	Positive	Negative	P-value
TRAV1-2	GMM	10	86	7438	249848	0.0005
TRAV8-6	SGL	6	121	5428	251858	0.053
TRAV13-2	SGL	2	125	2514	254772	0.35
TRAV21	SGL	8	119	7394	249892	0.031
TRAV19	SGL	8	119	10745	246541	0.26

Supplementary Table 3. Enrichment of V Genes Among SGL-CD1b and GMM-CD1b tetramer-sorted cells. The count of recovered TCRs using the specified V gene is reported from both patients studied, from GMM-CD1b or SGL-CD1b tetramer-sorted cells (TCRs from GMM-CD1b sorted cells = 96, TCRs from SGL-CD1b sorted cells = 127). IMGT nomenclature is used for variable (V) gene identification. Bulk T cell counts are reported as the sum of the templates from both patients that use the specified V gene using previously published ImmunoSEQ data from the same patients studied here[2]. Template counts were used to populate a 2x2 contingency table and unadjusted p-values resulting from a Fisher's exact test are shown.

Feature	p-value	Adjusted p-value
BCL6	0.0008633322	0.01812998
CTLA4	0.0774295439	0.23228863
EOMES	0.0157129218	0.08249284
FOXP3	0.0895233478	0.23499879
GATA3	0.6855045639	0.89972474
GZMB	0.2702441799	0.43654829
IFNG	0.0412465963	0.17323570
IL10	0.4903846154	0.73557692
IL12A	0.0543482280	0.19021880
IL13	1.0000000000	1.00000000
IL17A	1.0000000000	1.00000000
IL2	1.0000000000	1.00000000
MKI67	0.1457037759	0.33997548
PDCD1	1.0000000000	1.00000000
PRF1	0.0060037481	0.04202624
RORC	0.5617481485	0.78644741
RUNX1	0.7755665553	0.95805280
RUNX3	0.2600593808	0.43654829
TBET	0.0026350284	0.02766780
TGFB1	0.2380507842	0.43654829
TNF	0.1649880906	0.34647499

Supplementary Table 4. Differentially expressed genes among CD4 and CD8 CD1b-restricted T cells. P-values for each gene are summarized here P-values were calculated for each gene (Feature) between CD4 and CD8 T cells using following a Fisher's Exact Test (p-value) and Benjamini-Hochberg correction (adjusted p-value) (n = 23 features).

Gene	Reaction 1: Forward Primer	Reaction 1: Reverse Primer
PDCD1	TGACAGAGAGAAGGGCAGAAGT	GAAATCCAGCTCCCCATAGTC
CTLA4	CTTGCCTTGGATTTCAGCGG	TAGGTTGCCGCACAGACTTC
MKI67	CAAGACCCCAGTGAAGGAGC	CTGGGCGTTTTTGCTACGT
EOMES	TGTTCGTAGAGGTGGTGCTG	ATGCAGTCGGGGTTGGTATT
CD4	CCTCCCCTAAGCTGATGCTG	CGGCACCTGACACAGAAGAA
CD8A	AAATGCGAAATCAGGCTCCG	ATGATGGAGTTGCTCAGGGC
GATA3	GACGCGGCGCAGTACCCGCT	GGAGAAGGGGCTGAGATTCCAG
TBET	GCCTGTACGTCCACCCGGACT	CTGGGTTTCTTGGAAAGTAAAGATAT
FOXP3	GGCTCCTGCTGCATCGTAGCTGCT	GTCCGCTGCTTCTCTGGAGCCT
RORC	CCCGGGAGGAAGTGACTGGCTA	CCATGCCACCGTATTTGCCTTCAA
RUNX1	CCGCAGCATGGTGGAGGTGCT	GGTCATTAAATCTTGCAACCTGGTT
RUNX3	GCGCTCGATGGTGGACGTGCT	CGTTGAACCTGGCCACCTGGTT
BCL6	GCCAAACCAGAGGGGCCTGAG	GAGAGCCGCAGGACGTGCACTT
IL2	CTCACATTTAAGTTTTACATGCCCAA	GACAAAAGGTAATCCATCTGTTCAG
IL10	CCAGTTTTACCTGGAGGAGGTGA	GTAGGCTTCTATGTAGTTGATGAAGA
IL12A	GGGAGTTGCCTGGCCTCCAGAA	CGGTTCTTCAAGGGAGGATTTTTGT
IL13	CCCAGAACCAGAAGGCTCCGCT	CCCTCGCGAAAAAGTTTCTTTAAAT
IL17A	GACAAGAACTTCCCCCGGACTG	GGACCAGGATCTCTTGCTGGAT
IFNG	GGCTTTTCAGCTCTGCATCGTTTT	GGATGCTCTGGTCATCTTTAAAGTT
TNFA	CATGATCCGGGACGTGGAGCT	GGGCTACAGGCTTGTCACTCG
TGFB	GCATATATATGTTCTTCAACACATCA	CCCTCCACGGCTCAACCACT
PERFORIN	GTGTCTGTGGCCGGCTCACAC	CCGATATGCGGCCACCCAGCT
GRANZYMEB	GGGAAGCTCCATAAATGTCACCTT	GTTTTCCCAGGGGGGCCGTCT

Supplementary Table 5. Gene-specific primers used for transcriptional profiling reaction 1.

GENE	Reaction 2: Forward Primer	Reaction 2: Reverse Primer
PDCD1	CCAGGGTTTTCCCAGTCACGACA GGGTGACAGAGAGAAGGGCAGA	AGCGGATAACAATTTCACACAGGACGG TGCGCCTGGCTCCTATTG
CTLA4	CCAGGGTTTTCCCAGTCACGACT GCCTTGGATTTCAGCGGCAC	AGCGGATAACAATTTCACACAGGACGG ACCTCAGTGGCTTTGCC
MKI67	CCAGGGTTTTCCCAGTCACGACC CCCAGTGAAGGAGCAACCG	AGCGGATAACAATTTCACACAGGAAAG GGAGGGCTTGCAGAGCAT
EOMES	CCAGGGTTTTCCCAGTCACGACT GACCTGTGGCAAAGCCGAC	AGCGGATAACAATTTCACACAGGAGCA GTCGGGGTTGGTATTTGTG
CD4	CCAGGGTTTTCCCAGTCACGACT CTCGAAGCGGGAGAAGGCG	AGCGGATAACAATTTCACACAGGAAGC CCAATGAAAAGCAGGAGGC
CD8A	CCAGGGTTTTCCCAGTCACGACC TTCGAGCCAAGCAGCGTCC	AGCGGATAACAATTTCACACAGGACTG GAAGAGCCACGAGCAGC
GATA3	CCAGGGTTTTCCCAGTCACGACG CCGGAGGAGGTGGATGTGCTT	AGCGGATAACAATTTCACACAGGAGGG GAGGCGGTGTGGTGGCT
TBET	CCAGGGTTTTCCCAGTCACGACC CCAACACAGGAGCGCACTGG	AGCGGATAACAATTTCACACAGGACGT GTTGGAAGCGTTGCAGGCT
FOXP3	CCAGGGTTTTCCCAGTCACGACG GCAGCCAAGGCCCTGTCGT	AGCGGATAACAATTTCACACAGGACCA GGATGGCCCAGCGGATGA
RORC	CCAGGGTTTTCCCAGTCACGACA GAGGAAGTCCATGTGGGAGATGT	AGCGGATAACAATTTCACACAGGATCA GCATTGTAGGCCCGGCACATC
RUNX1	CCAGGGTTTTCCCAGTCACGACG CGAGCTGGTGCGCACCGACA	AGCGGATAACAATTTCACACAGGAGGC TGCGGTAGCATTTCTCAGCT
RUNX3	CCAGGGTTTTCCCAGTCACGACG GACCACGCAGGCGAGCTCGT	AGCGGATAACAATTTCACACAGGACGG CCGAGGCATTGCGCAGCT
BCL6	CCAGGGTTTTCCCAGTCACGACC CTACACGGCCCCACCTGCCT	AGCGGATAACAATTTCACACAGGAGGG TGCATGTAGAGTGGTGAGTG
IL2	CCAGGGTTTTCCCAGTCACGACC CACAGAACTGAAACATCTTCAGT	AGCGGATAACAATTTCACACAGGATTC TACAATGGTTGCTGTCTCA
IL10	CCAGGGTTTTCCCAGTCACGACC CCAAGCTGAGAACCAAGACCCA	AGCGGATAACAATTTCACACAGGAGTC AAACTCACTCATGGCTTTGTA
IL12A	CCAGGGTTTTCCCAGTCACGACA GACCTCTTTTATGATGGCCCTGT	AGCGGATAACAATTTCACACAGGAGGC ACAGTCTCACTGTTGAAATTCA
IL13	CCAGGGTTTTCCCAGTCACGACG GTATGGAGCATCAACCTGACAG	AGCGGATAACAATTTCACACAGGAGGT CCTTTACAAACTGGGCCAC
IL17A	CCAGGGTTTTCCCAGTCACGACC AACCTGAACATCCATAACCGGAA	AGCGGATAACAATTTCACACAGGAGGG GACAGAGTTCATGTGGTAGT
IFNG	CCAGGGTTTTCCCAGTCACGACG GGTTCTCTTGGCTGTTACTGC	AGCGGATAACAATTTCACACAGGAGTT TGAAGTAAAAGGAGACAATTTG
TNFA	CCAGGGTTTTCCCAGTCACGACG GAGGCGCTCCCCAAGAAGAC	AGCGGATAACAATTTCACACAGGACGA GAAGATGATCTGACTGCCTG
TGFB	CCAGGGTTTTCCCAGTCACGACC CGAGAAGCGGTACCTGAACC	AGCGGATAACAATTTCACACAGGACCG CACAACTCCGGTGACATCA

PERFORIN	CCAGGGTTTTCCCAGTCACGACG CCAACTTTGCAGCCCAGAAGA	AGCGGATAACAATTTCACACAGGAGGG TGCCGTAGTTGGAGATAAG
GRANZYMEB	CCAGGGTTTTCCCAGTCACGACC CACAATATCAAAGAACAGGAGCC	AGCGGATAACAATTTCACACAGGAGCC ACACTGCATGTCTGCCCT

Supplementary Table 6. Gene-specific primers used for transcriptional profiling reaction 2.

Supplementary Figure 1.

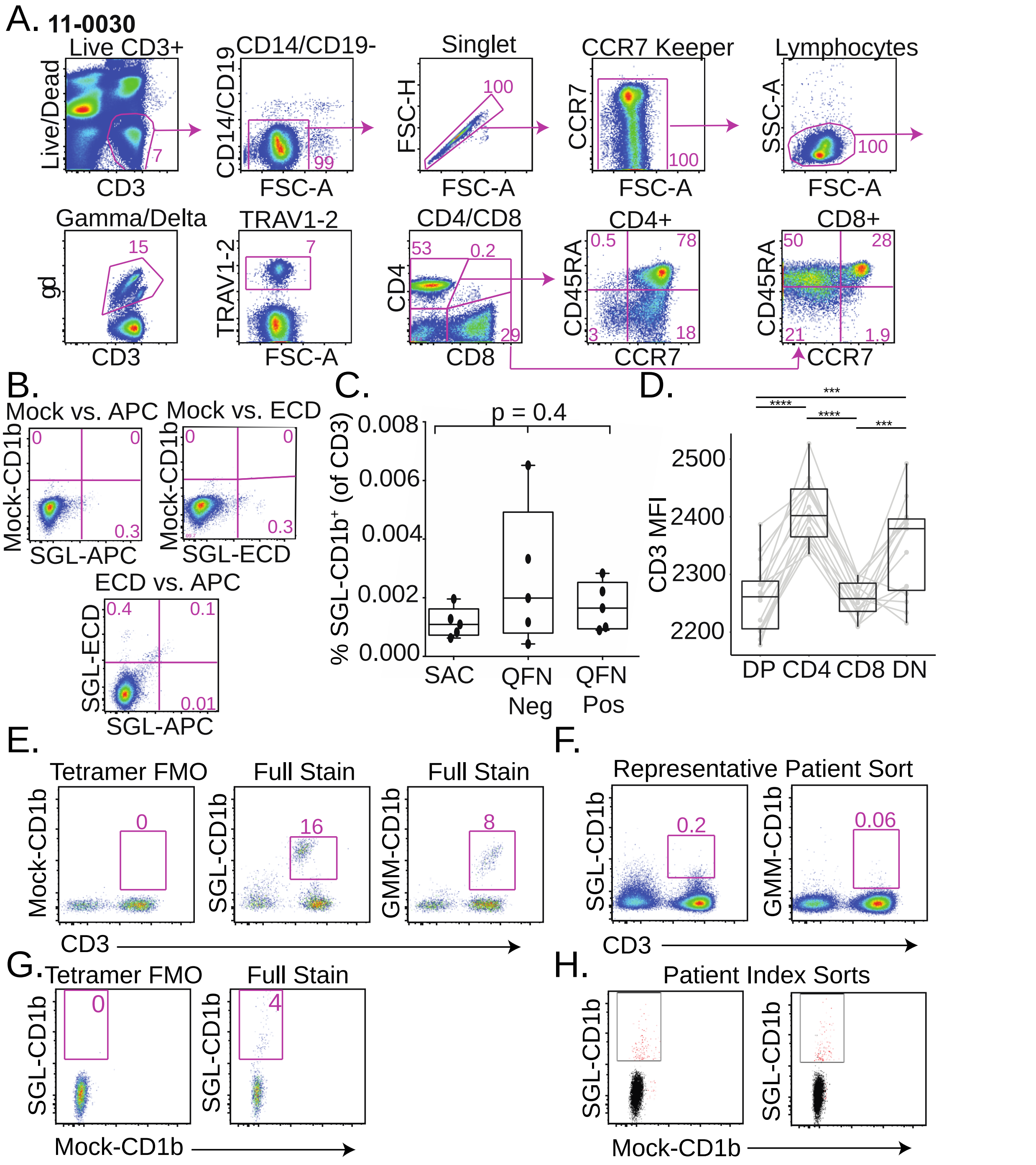

Supplementary Figure 1. Gating Strategy for Flow Cytometry and Cell Sorting. (A) Representative gating strategy for identification of SGL-specific T cells. The gates proceeded from Live and CD3$^+$ cells to CD14$^-$ and CD19$^-$ cells to single cells to a CCR7 keeper gate to lymphocytes by size gating. The CCR7 Keeper Gate is applied to eliminate events stained with dye aggregates, as these can interfere with downstream analysis and gating of rare cell populations[3]. After this point, gates were drawn for γδ T cells, TRAV1-2, CD4 and CD8, and CD45RA and CCR7 independently for CD4 and CD8 T cells. (B) Gates were drawn for SGL-CD1b tetramer-positive cells as defined by dual staining with two SGL-CD1b

tetramers, and negative for Mock-loaded CD1b tetramer. (C) Boxplots depict the minimum and maximum as the smallest and largest number of the dataset, excluding outliers, the median and interquartile range of SGL-CD1b frequency, expressed as a percentage of $CD3^{+}$ T cells. Each dot represents the percentage of SGL-CD1b-specific T cells from one donor. (Kruskal Wallis with Dunn post-test, $p = 0.4$, $n = 15$). (D) Boxplots depict the minimum and maximum as the smallest and largest number of the dataset, excluding outliers, the median and interquartile range of CD3 MFI of all T cells within each co-receptor group (double positive (DP), CD4, CD8, and double negative (DN)). Each dot represents the percent of one sample. The percent of cells in each group was compared to that present in total CD3+ T cells (grey). (Two-sided Friedman test with Dunn post-test, *** = $p < 0.0001$, ** = $p = 0.004$, $n = 15$). (E) Natural SGL-CD1b and GMM-CD1b tetramers were incorporated into a multi-parameter flow cytometry assay to isolate SGL-specific and GMM-specific T cells using fluorescence activated cell sorting (FACS). The tetramer positive gate was defined by a Mock-loaded CD1b negative control tetramer (left) and a positive control using SGL- and GMM-specific T cell lines diluted in donor PBMC (middle, right). (F) Natural SGL-CD1b and GMM-CD1b tetramer positive T cells in the blood were sorted from cryopreserved PBMC donated by South African adults with new diagnosis of active TB disease ($n = 2$). (G) Gates were drawn for SGL-CD1b tetramer-positive cells as defined by staining with SGL-CD1b tetramer and negative for mock-loaded CD1b tetramer. To define these gates, we included an SGL-CD1b fluorescence minus one (FMO) control (left), and a T cell line positive control (right), to ensure the gates reliably captured cells that stain with SGL-CD1b tetramer. In this experiment, we included an anti-streptavidin antibody conjugated to the same fluorochrome as the SGL-CD1b tetramer to increase the tetramer MFI. (H) SGL-CD1b tetramer positive T cells in the blood were single-cell sorted from cryopreserved PBMC donated by South African adults with new diagnosis of active TB disease ($n = 2$). This single cell sort was also indexed, meaning that the MFIs for each channel were saved for each single cell, and this information was combined with the TCR assignments to assign a co-receptor group to a cell. The negative population (black) is visualized here using a no tetramer control for each individual (TB-1124, left; TB-1127, right). The sorted cells (red) are visualized within the sort gate ($n = 184$ per donor).

Supplementary Figure 2.

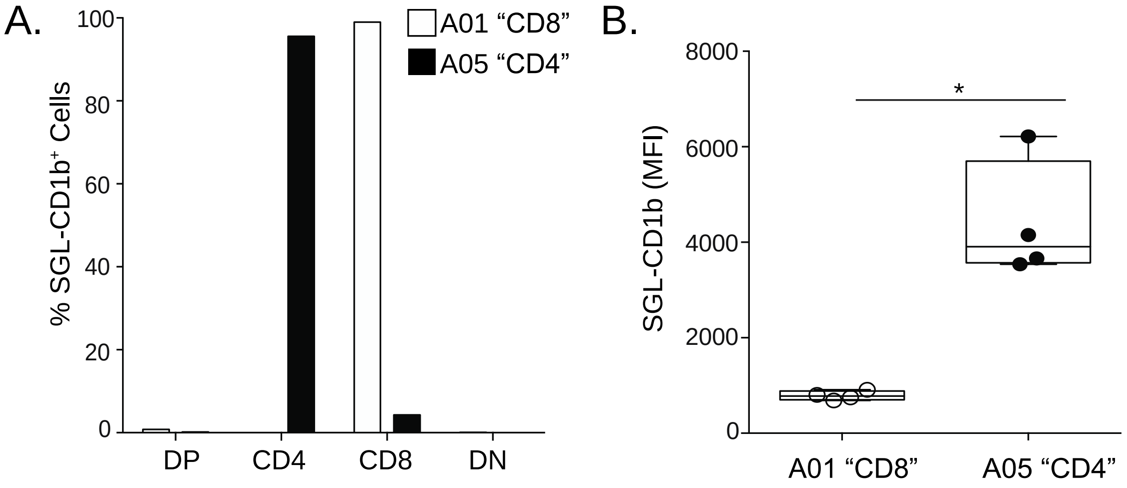

Supplementary Figure 2. Differences in functional avidity between SGL-specific T cell lines. A01 and A05 T cell lines are specific for SGL and examined by multicolor flow cytometry. (A) Tetramer-positive cells within the A05 T cell line express CD4, and lack CD8 and CD8 expression (black). Tetramer-positive cells within the A01 T cell line express CD8 and CD8, but lack CD4 expression (white). (B) Boxplots depict the minimum and maximum as the smallest and largest number of the dataset, excluding outliers, the median and interquartile range of the SGL-CD1b MFI of A01 (white) and A05 (black) from four independent experiments. (Two-sided Mann-Whitney, $p = 0.028$, $n = 4$).

Supplementary Figure 3.

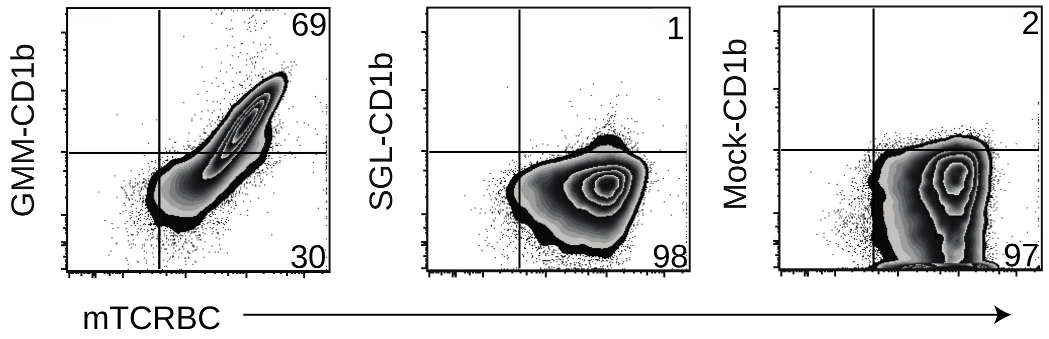

Supplementary Figure 3. **Specificity of staining with SGL-CD1b tetramer.** Jurkat cells were transduced with the germline-encoded mycolyl reactive (GEM) TCR (clone 42)[4,5]. Jurkat cells transduced with the GEM TCR stain reliably with glucose monomycolate (GMM)-CD1b tetramer and a murine TCR-β chain constant region (mTCRBC) specific antibody (left). These GMM-specific Jurkat cells do not bind SGL-CD1b or mock-loaded CD1b tetramer (middle, right).

Supplementary Figure 4.

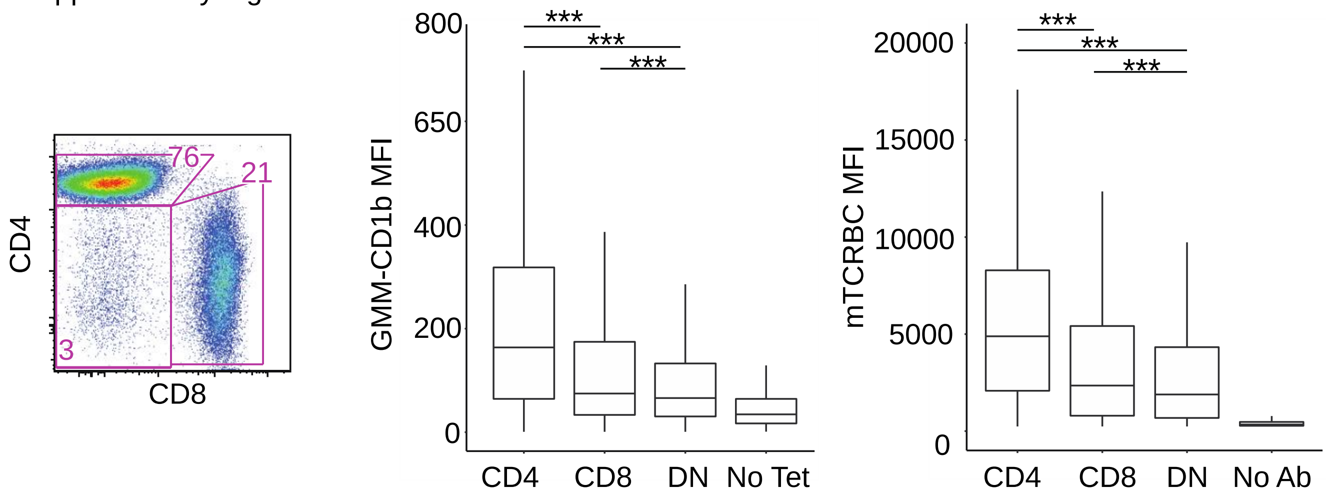

Supplementary Figure 4. Functional avidity of CD4, CD8, and DN T cells transduced with a GMM-specific TCR. Flow plot depicts the percent of transduced T cells that are CD4, CD8, or DN. Boxplot depicts the median and interquartile range of the GMM-CD1b or mTCRBC MFI of each CD4, CD8, and DN T cell that was transduced with the GEM TCR. The CD4+ transduced T cells stained with GMM-CD1b tetramer with a 1.29-fold higher MFI than the CD8 GMM-specific T cells, and a 2.00-fold higher MFI than DN transduced T cells (Two-way ANOVA, post-hoc Dunn test, *** = $p < 0.0001$, n = 107,935). The CD4+ transduced T cells also stained with the anti-murine TCR-β chain constant region (mTCRBC) antibody with a 1.31-fold higher MFI then CD8+ T cells and a 1.53-fold higher MFI than DN T cells ($p < 0.0001$ and $p < 0.0001$, ANOVA with Tukey post-test). The control populations (No Tet and No Ab) were not included in the statistical analysis. Data are representative of two independent rounds of primary T cell transduction with the GEM TCR.

Supplementary Figure 5.

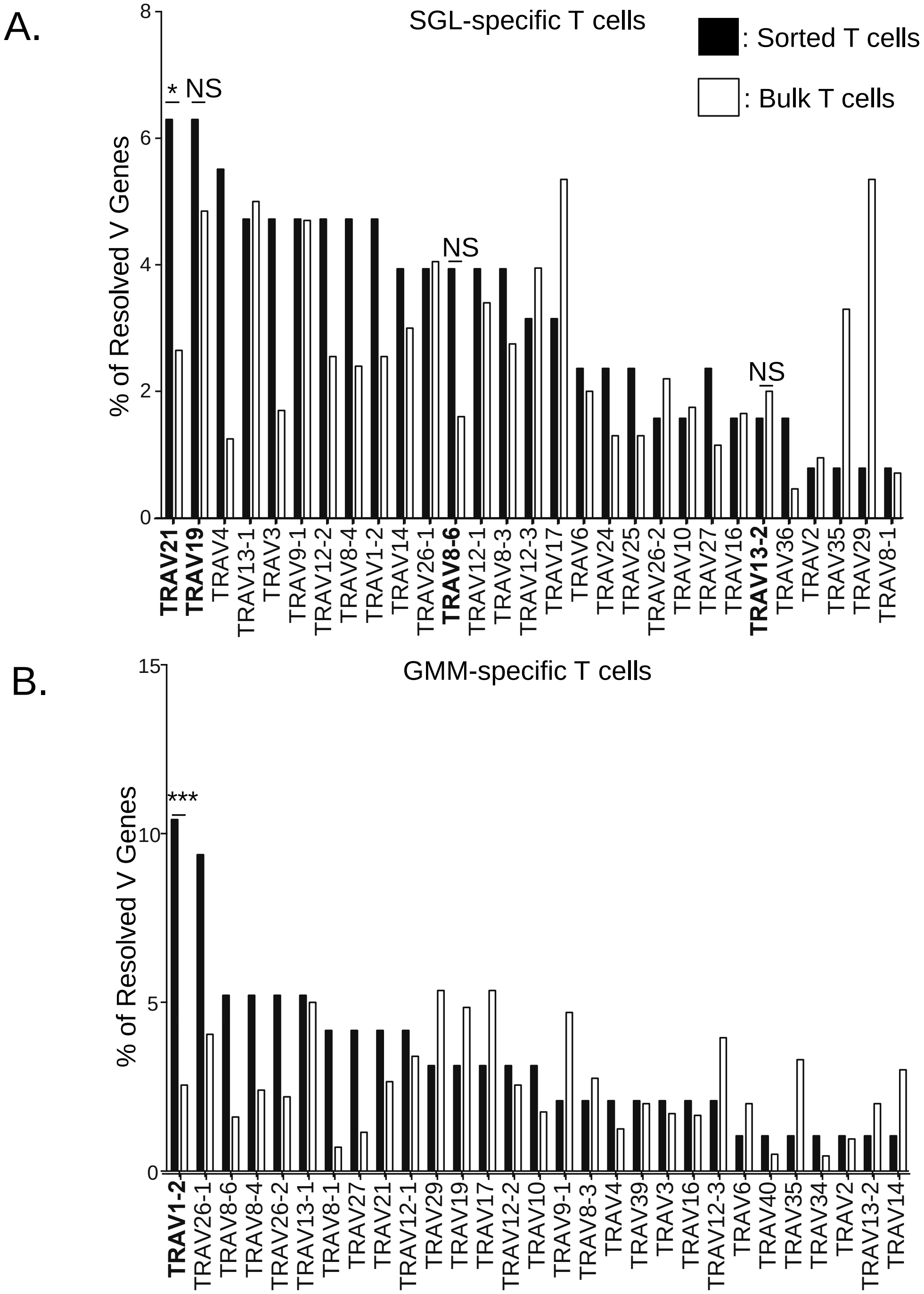

Supplementary Figure 5. Glycolipid-specific T cells express a diverse TCR repertoire Variable (V) genes from recovered TCRs ordered by decreasing prevalence in the dataset. For tetramer-sorted cells, V genes were assigned using VDJFasta using IMGT nomenclature. For bulk T cells, V genes were identified using the ImmunoSEQ TCR α assay (Adaptive Biotechnologies). This data set was previously published, and only the two relevant patient samples are analyzed here[2]. Bar plot depicts the percentage of total recovered T cell receptor variable (V) genes identified from (A) SGL-CD1b tetramer-sorted cells from participant TB-1117 (n = 72) and participant TB-1119 (n = 55) (black), compared to the percentage

of bulk T cells that utilize that particular V gene (white). (* = p = 0.031, NS = p > 0.05, Two-sided Fisher's Exact Test). (B) Bar plot depicts the percentage of total recovered T cell receptor variable (V) genes identified from GMM-CD1b tetramer-sorted cells from participants TB-1117 (n = 30) and TB-1119 (n = 66) (black), compared to the percentage of bulk T cells that utilize that particular V gene (white) (*** = p = 0.0005, Two-sided Fisher's Exact Test).

Supplementary Figure 6.

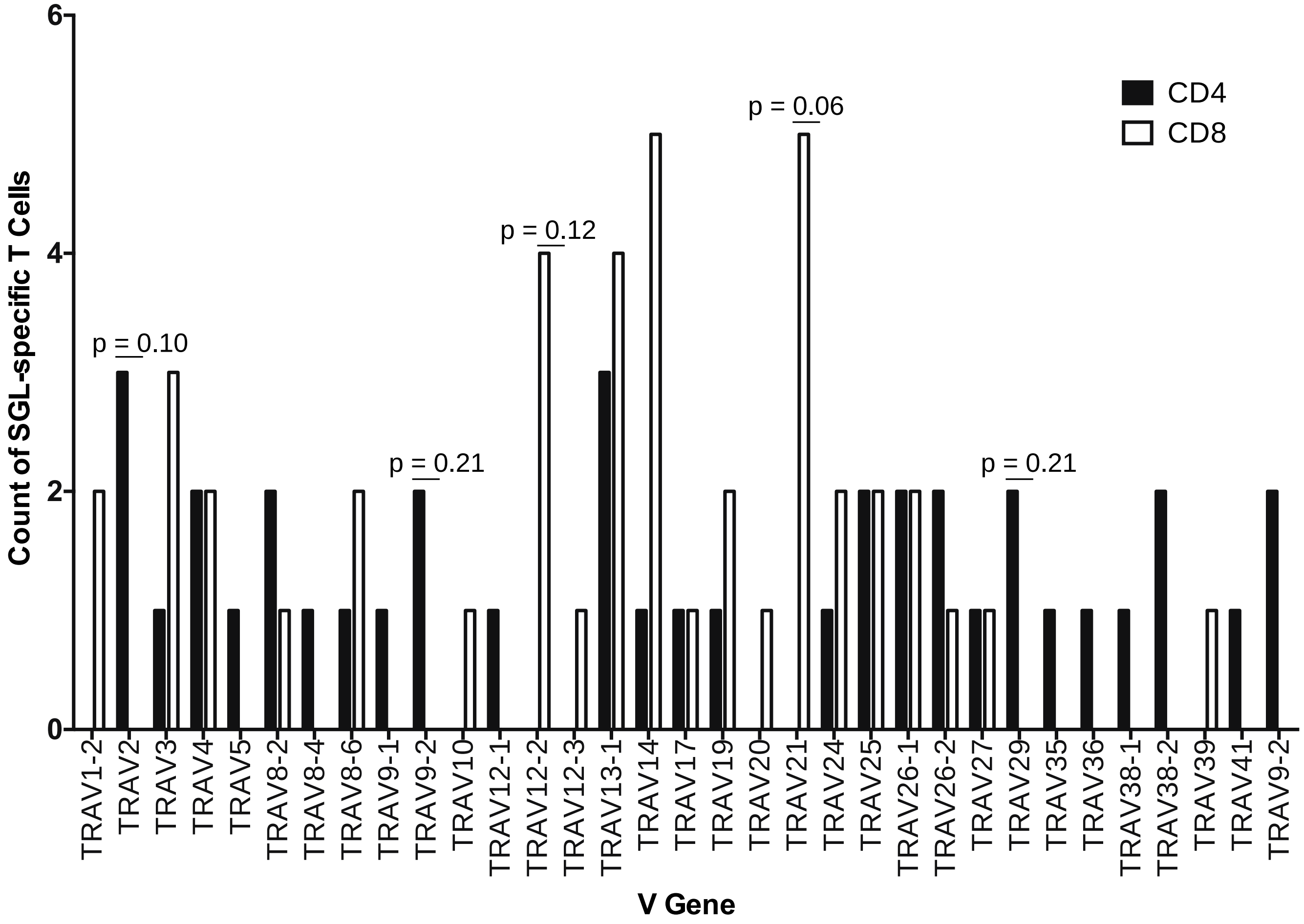

Supplementary Figure 6. TCR-α chain V gene usage by CD4 and CD8 SGL-specific T cells. Variable (V) genes from recovered CD4 (black) and CD8 (white) TCRs ordered numerically. For tetramer-sorted cells, V genes were assigned using VDJFasta using IMGT nomenclature. Bar plot depicts the percentage of total recovered T cell receptor variable (V) genes identified from SGL-CD1b tetramer-sorted cells from participants TB-1117 (n = 24), TB-1119 (n = 18), TB-1124 (n = 21), and TB-1127 (n = 19). Co-receptor expression was defined by mRNA in participants TB-1117 and TB-1119. In participants TB-1124 and TB-1127, the tetramer-positive cells were index sorted into the 96-well PCR plate, and CD4 and CD8 expression was defined by staining with anti-CD4 and anti-CD8 antibodies. P-values stated are from Two-sided Fisher’s exact tests comparing V gene usage in CD4 and CD8 T cells and are unadjusted.

Supplementary Figure 7.

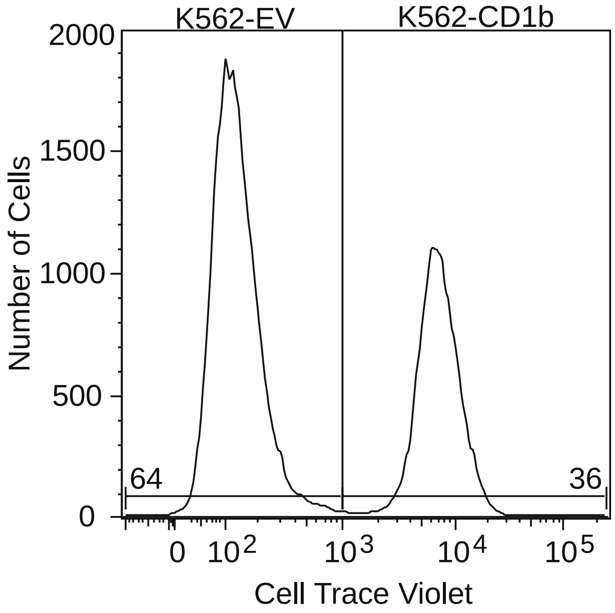

Supplementary Figure 7. Control populations for cytotoxicity assay. To aid in our cytotoxicity calculation in Figure 5D, we quantified the ratio of K562-CD1b and K562-EV cells in culture. K562-EV and K562-CD1b antigen presenting cells were labeled “low” and “high” with Cell Trace Violet, respectively, and mixed in a 1:1 ratio. These cells were not co-cultured with T cells or lipid antigen. This control enables us to quantify changes in the frequency of K562-CD1b cells that are unrelated to T cell cytotoxic activity to ensure that our “% Cell Death” calculation accurately reflects the reduction of K562-CD1b cells that results from co-culturing with T cells and antigen.

SUPPLEMENTARY REFERENCES

1. Mahomed, H. *et al.* Predictive factors for latent tuberculosis infection among adolescents in a high-burden area in South Africa. *Int. J. Tuberc. Lung Dis.* **15,** 331–6 (2011).
2. DeWitt, W. S. *et al.* A Diverse Lipid Antigen–Specific TCR Repertoire Is Clonally Expanded during Active Tuberculosis. *J. Immunol.* (2018). doi:10.4049/jimmunol.1800186
3. Layton, E. D. *et al.* Validation of a CD1b tetramer assay for studies of human mycobacterial infection or vaccination. *J. Immunol. Methods* **458,** 44–52 (2018).
4. Gras, S. *et al.* T cell receptor recognition of CD1b presenting a mycobacterial glycolipid. *Nat. Commun.* **7,** 13257 (2016).
5. Van Rhijn, I. *et al.* A conserved human T cell population targets mycobacterial antigens presented by CD1b. *Nat. Immunol.* **14,** 706–13 (2013).
