# Supplementary material for: CD4 and CD8 co-receptors modulate functional avidity of CD1b-restricted T cells
Source: Nat Commun. 2022 Jan 10;13:78. doi: 10.1038/s41467-021-27764-w (PMC8748927; doi:10.1038/s41467-021-27764-w)
Supplement: Supplementary file 3 — Reporting summary [file 41467_2021_27764_MOESM3_ESM.pdf]

nature research

Corresponding author(s): Chetan Seshadri

Last updated by author(s): October 26, 2021

Reporting Summary

Nature Research wishes to improve the reproducibility of the work that we publish. This form provides structure for consistency and transparency in reporting. For further information on Nature Research policies, see our Editorial Policies and the Editorial Policy Checklist.

Statistics

For all statistical analyses, confirm that the following items are present in the figure legend, table legend, main text, or Methods section.

n/a	Confirmed	
☐	☒	The exact sample size (n) for each experimental group/condition, given as a discrete number and unit of measurement
☐	☒	A statement on whether measurements were taken from distinct samples or whether the same sample was measured repeatedly
☐	☒	The statistical test(s) used AND whether they are one- or two-sided<br>*Only common tests should be described solely by name; describe more complex techniques in the Methods section.*
☒	☐	A description of all covariates tested
☐	☒	A description of any assumptions or corrections, such as tests of normality and adjustment for multiple comparisons
☐	☒	A full description of the statistical parameters including central tendency (e.g. means) or other basic estimates (e.g. regression coefficient) AND variation (e.g. standard deviation) or associated estimates of uncertainty (e.g. confidence intervals)
☐	☒	For null hypothesis testing, the test statistic (e.g. F, t, r) with confidence intervals, effect sizes, degrees of freedom and P value noted<br>*Give P values as exact values whenever suitable.*
☒	☐	For Bayesian analysis, information on the choice of priors and Markov chain Monte Carlo settings
☒	☐	For hierarchical and complex designs, identification of the appropriate level for tests and full reporting of outcomes
☒	☐	Estimates of effect sizes (e.g. Cohen's d, Pearson's r), indicating how they were calculated

Our web collection on statistics for biologists contains articles on many of the points above.

Software and code

Policy information about availability of computer code

Data collection	BD FACSDiva v9.0
Data analysis	Commercial: FlowJo version 9.9.6 GraphPad Prism version 6.0, Open-source: R version 3.5.2, OpenCyto (opencyto.org), VDJFasta (https://github.com/immunoengineer/vdjfasta), Seurat Pipeline (https://satijalab.org/seurat/). Published manuscripts for the above open-source software are included in the References of this study. Our data analysis workflow is available at: https://github.com/seshadrilab (DOI: 10.5281/zenodo.5542410).

For manuscripts utilizing custom algorithms or software that are central to the research but not yet described in published literature, software must be made available to editors and reviewers. We strongly encourage code deposition in a community repository (e.g. GitHub). See the Nature Research guidelines for submitting code & software for further information.

Data

Policy information about availability of data

All manuscripts must include a data availability statement. This statement should provide the following information, where applicable:

- Accession codes, unique identifiers, or web links for publicly available datasets
- A list of figures that have associated raw data
- A description of any restrictions on data availability

Raw data are published as Source Data of this manuscript and available on a GitHub repository (DOI: 10.5281/zenodo.5542410) . Public database IMGT was used to map TCR sequences and can be accessed here: https://www.imgt.org/

Field-specific reporting

Please select the one below that is the best fit for your research. If you are not sure, read the appropriate sections before making your selection.

[x] Life sciences   [ ] Behavioural & social sciences   [ ] Ecological, evolutionary & environmental sciences

For a reference copy of the document with all sections, see nature.com/documents/nr-reporting-summary-flat.pdf

Life sciences study design

All studies must disclose on these points even when the disclosure is negative.

Sample size	No sample size calculation was performed. Sample size was selected based on previous cohort knowledge and data analysis plans.
Data exclusions	No data were excluded from analysis. Pre-specified exclusion criteria were established, and no samples met specified thresholds for poor viability (CD3+ Event count < 10,000 cells or CD4 Event count < 3,000 cells).
Replication	Number of biological and technical replicates are specified in figure legend, and all replicates where stated were successful. Data in Figures 1, 5, and 7 were not validated through biological or technical replicates due to sample limitations and cost of study and were instead validated through alternative experimental approaches.
Randomization	Samples were not randomized into experimental batches as data included in Figure 1 as all samples were acquired in one batch (acquired on the same day with same flow cytometer parameters). No randomization or confounder control was required because each individual served as their own control (paired analyses).
Blinding	Individual who acquired samples for Figure 1 was blinded to disease status group allocation and demographic information during sample preparation and data acquisition.

Reporting for specific materials, systems and methods

We require information from authors about some types of materials, experimental systems and methods used in many studies. Here, indicate whether each material, system or method listed is relevant to your study. If you are not sure if a list item applies to your research, read the appropriate section before selecting a response.

Materials & experimental systems

n/a	Involved in the study	
[ ]	[x]	Antibodies
[ ]	[x]	Eukaryotic cell lines
[x]	[ ]	Palaeontology and archaeology
[x]	[ ]	Animals and other organisms
[ ]	[x]	Human research participants
[x]	[ ]	Clinical data
[x]	[ ]	Dual use research of concern

Methods

n/a	Involved in the study	
[x]	[ ]	ChIP-seq
[ ]	[x]	Flow cytometry
[x]	[ ]	MRI-based neuroimaging

Antibodies

Antibodies used

Specificity	Purpose	Fluorochrome	Clone	Supplier	Catalog Number	Dilution
CD3	Lineage	BUV395	UCHT1	BD Biosciences	563548	1:50
CD4	Lineage	APC H7	13B8.2	BD Biosciences	560837	1:50
CD8β	Lineage	BB700	2ST8.5H7	BD Biosciences	745761	1:10
CD45RA	Memory	BUV737	HI100	BD Biosciences	564442	1:500
CCR7	Memory	BV711	150503	BD Biosciences	566602	1:100
Pan-γδ	γδ T cells	PE-Vio770	11F2	Miltenyi Biotec	130-099-762	1:50
Vδ2	Vγ9δ2 T cells	AF700	B6	BioLegend	331416	1:100
TRAV1-2	TCR Identification	BV605	3C10	BioLegend	351720	1:25
CD14	Exclusion	V785	M5E2	BioLegend	301840	1:50
CD19	Exclusion	V785	SJ25C1	BioLegend	363028	1:50
Green Dead Cell Stain	Viability	FITC	N/A	Life Technologies	L23101	1:1000
CD3	Lineage	ECD	UCHT1	Beckman Coulter	IM2705U	1:50
CD4	Lineage	APC Ax750	13B8.2	Beckman Coulter	A94685	1:50
CD8a	Lineage	PerCP Cy5.5	SK1	BD Biosciences	341051	1:10
IFN-γ	Th1	V450	B27	BD Biosciences	560371	1:50
TNF	Th1	FITC	MAb11	BD Biosciences	554512	1:50

IL-2	Th1	PE	MQ1-17H12	BD Biosciences	559334	1:10
IL-4	Th2	APC	MP4-25D2	BD Biosciences	554486	1:250
CD154	CD4 Activation/ B cell help	PE Cy5	TRAP1	BD Biosciences	555701	1:5
CD107a	Degranulation	PE Cy7	H4A3	BD Biosciences	561348	1:50
Aqua Live/ Dead Stain	Viability	V510	N/A	Life Technologies	L34966	1:100
mTCRBC	Exogenous TCR Identification	APC	H57597	BD Biosciences	553174	1:50
CD28/CD49d	Activation	Unconjugated	Not Listed	BD Biosciences	347690	1:25

Validation

All antibodies are titered for optimal staining on human peripheral blood mononuclear cells (PBMC) prior to use to ensure reactivity and determine optimal staining volume. This information is included in Supplemental Table 2. Antibodies were validated for reactivity by comparing staining of antibodies in human PBMC to known staining patterns. anti-murine TCR beta chain constant region antibody is validated on Jurkat cell lines expressing a modified TCR that contains a murine TCR constant region. This reagent was validated by staining a cell line transduced with a TCR with a murine constant region.

Eukaryotic cell lines

Policy information about cell lines

Cell line source(s)

Jurkat cells were provided by Dr. Thomas Blankenstein (Max Delbrück Center for Molecular Medicine, Berlin, Germany). K562 cells were provided by Dr. D. Branch Moody (Brigham and Women's Hospital, Boston, MA). Lenti-X HEK 293T cells were purchased from Clontech (Mountain View, CA). Upon receipt, Jurkat and K562 cell lines were validated by flow cytometry for purity and expression of markers of interest. The original commercial source of the cell lines is unknown, and both Jurkat and K562 cell lines have been significantly modified prior to our receipt.

Authentication

No new cell lines are described in this study. Cells received from collaborators were validated only for expression of genes of interest using flow cytometry.

Mycoplasma contamination

Cell lines tested negative for Mycoplasma contamination on Nov 29, 2018 using an enzyme-based assay, and these cells were then cryopreserved and thawed to generate these data.

Commonly misidentified lines
(See ICLAC register)

Cell lines used in this study are not included in this register.

Human research participants

Policy information about studies involving human research participants

Population characteristics

Age, sex and disease status is described for each South African adolescent sample in Supplemental Table 1. Demographic information is not available for U.S. healthy controls.

Recruitment

First, U.S. healthy controls were recruited and enrolled at the Seattle HIV Vaccine Trials Unit as part of a cohort of healthy adults used to provide blood samples for developing and testing new assays. Second, we studied a subset of 6363 South African adolescents that were enrolled into a study that aimed to determine the incidence and prevalence of tuberculosis infection and disease in South Africa. Adolescents were enrolled at eleven high schools in the Worcester region of the Western Cape of South Africa. Individuals self-selected to be enrolled in these studies, which may introduce bias from the population at large.

Ethics oversight

The study protocols were approved by the IRBs of the University of Washington, the Fred Hutchinson Cancer Research Center, and the University of Cape Town. Written informed consent was obtained from all adult participants as well as from the parents and/or legal guardians of the adolescents who participated. In addition, written informed assent was obtained from the adolescents.

Note that full information on the approval of the study protocol must also be provided in the manuscript.

Flow Cytometry

Plots

Confirm that:

- [x] The axis labels state the marker and fluorochrome used (e.g. CD4-FITC).
- [x] The axis scales are clearly visible. Include numbers along axes only for bottom left plot of group (a 'group' is an analysis of identical markers).
- [x] All plots are contour plots with outliers or pseudocolor plots.
- [x] A numerical value for number of cells or percentage (with statistics) is provided.

Methodology

Sample preparation

For ex vivo analysis of SGL-CD1b tetramer positive cells, we studied two cohorts of healthy participants. First, U.S. healthy

	controls were recruited and enrolled at the Seattle HIV Vaccine Trials Unit as part of a cohort of healthy adults who to provided blood samples for developing and testing new assays. Peripheral blood mononuclear cells (PBMC) were collected by leukapheresis from five HIV-seronegative individuals with a known T cell response to cytomegalovirus (CMV) were used here. We also utilized cryopreserved PBMC samples donated by adults with a new diagnosis of active tuberculosis also from the Worcester region of the Western Cape of South Africa.
Instrument	BD LSR Fortessa, BD FACS Aria II
Software	BD FACSDiva, FlowJo v9.9.6
Cell population abundance	Cell sorting population abundance is relevant to Figures 5, 7. Cell population abundance described in Supplemental Figure 1. Post-sort purity was not evaluated due to sorting into 96-well plate. Figure 1 also involved identification of low-frequency cells, and gating strategy and identification is detailed in Supplemental Figure 1 and Figure 1A. Representative plot is shown in Figure 1B.
Gating strategy	Gating strategy for ex vivo identification of antigen-specific T cells described in Supplemental Figure 1.

☒ Tick this box to confirm that a figure exemplifying the gating strategy is provided in the Supplementary Information.
